# Astrocytic Igfbp2 Promotes Spontaneous Seizures in a Mouse Model of Mesial Temporal Lobe Epilepsy

**DOI:** 10.1002/glia.70099

**Published:** 2025-11-15

**Authors:** Shinichi Kinoshita, Nobuyoshi Matsumoto, Shota Morikawa, Yuji Ikegaya, Ryuta Koyama

**Affiliations:** ^1^ Laboratory of Chemical Pharmacology, Graduate School of Pharmaceutical Sciences The University of Tokyo Bunkyo‐ku Tokyo Japan; ^2^ Institute for AI and Beyond The University of Tokyo Tokyo Japan; ^3^ Laboratory of Molecular Neurobiology, Department of Biophysics and Biochemistry The University of Tokyo Bunkyo‐ku Tokyo Japan; ^4^ Center for Information and Neural Networks, National Institute of Information and Communications Technology Suita City Osaka Japan; ^5^ Department of Translational Neurobiology National Institute of Neuroscience National Center of Neurology and Psychiatry Kodaira Tokyo Japan

**Keywords:** astrocytes, epilepsy, excitability, glia–neuron interaction, hippocampus, Igfbp2, transcriptome

## Abstract

Mesial temporal lobe epilepsy (MTLE) is a common, frequently drug‐resistant epilepsy characterized by seizures arising from the hippocampus. Its hallmark pathology is hippocampal sclerosis with neuronal loss and reactive astrogliosis. Although astrocytes have emerged as potential targets for antiepileptic therapies, their role in epilepsy development remains poorly defined. Here, we combined adeno‐associated virus (AAV)‐mediated labeling with translating ribosomal affinity purification (TRAP) to generate astrocyte‐enriched transcriptome profiles from sclerotic hippocampal regions in a mouse model of MTLE. This analysis identified a marked upregulation of insulin‐like growth factor‐binding protein 2 (Igfbp2) in reactive astrocytes. Functional studies revealed that astrocytic Igfbp2 increases the excitability of dentate granule cells and promotes spontaneous recurrent seizures. These findings reveal Igfbp2 as a key astrocytic modulator of hippocampal excitability and identify it as a potential therapeutic target for epilepsy.

## Graphical abstract

- Igfbp2 is upregulated in hippocampal astrocytes of MTLE model mice.
- Igfbp2 enhances dentate granule cell excitability.
- Astrocytic Igfbp2 deletion reduces spontaneous seizures.

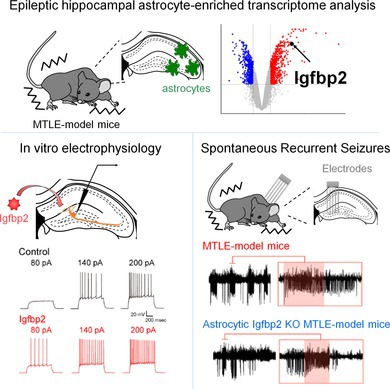

## 1 Introduction

Epilepsy is one of the most common chronic neurological disorders, characterized by recurrent seizures resulting from aberrant synchronous neuronal activity. Among its various subtypes, mesial temporal lobe epilepsy (MTLE) is a common and frequently drug‐resistant form, in which seizures arise from the hippocampus. Despite the availability of anti‐seizure medications, approximately 30% of MTLE patients experience poor seizure control. Most existing treatments primarily target neuronal ion channels, yet they fail to fully prevent seizure recurrence (Perucca and Mula 2013), highlighting the need for novel therapeutic targets and mechanisms of action. In particular, exploring the roles of glial cells may provide new insights into drug‐resistant epilepsies such as MTLE.

A hallmark pathology of MTLE is hippocampal sclerosis, defined by profound neuronal loss and reactive astrogliosis in the sclerotic hippocampus (Thom 2014). Astrocytes, the most abundant glial cell type in the brain, are essential regulators of brain homeostasis. They influence synaptic transmission and neuronal excitability by modulating extracellular potassium and glutamate concentrations and by releasing gliotransmitters, factors known to impact seizure dynamics (Bedner et al. 2015; Fernández‐García et al. 2020; Sano et al. 2021; Vezzani et al. 2022). Advances in astrocyte‐targeted manipulation techniques, such as optogenetics and chemogenetics, have further demonstrated their active roles in shaping neuronal network activity (Adamsky et al. 2018; Nagai et al. 2019).

Recent studies in rodent models have implicated astrocytes in acute seizure activity (Onodera et al. 2021; Zhao et al. 2022), but whether they contribute to the development of spontaneous recurrent seizures (SRSs), a hallmark of chronic epilepsy, remains poorly understood. Notably, few studies have demonstrated seizure suppression by targeting astrocytes, leaving their mechanistic involvement in epileptogenesis unresolved. To address this gap, we performed astrocyte‐enriched transcriptomic profiling of the epileptic hippocampus using translating ribosome affinity purification (TRAP) in combination with adeno‐associated virus (AAV)‐mediated gene delivery in a mouse model of MTLE. This approach enabled us to identify molecular signatures of reactive astrocytes in the sclerotic hippocampus. Our findings uncover astrocytic signals that may contribute to the emergence of SRSs and offer insights into astrocyte‐based therapeutic strategies for MTLE.

## 2 Material & Methods

### 2.1 Animals

In the present study, animal experiments were performed under the approval of the Animal Experiment Ethics Committee at the University of Tokyo (approval number: P4‐2, P4‐4, P4‐7) and in accordance with the guidelines for the care and use of laboratory animals of the University of Tokyo. C57BL/6J mice (SLC, Shizuoka, Japan) and Aldh1l1‐EGFP (MMRC, 011015) were housed in cages under standard laboratory conditions (12‐h light/dark cycle and free access to food and water). Aldh1l1‐EGFP mice were backcrossed with C57BL/6J mice. In this study, six‐week‐old male C57BL/6J mice and 6–8‐week‐old heterozygous male and female Aldh1l1‐EGFP mice were used. The mice used for LFP recording were housed individually after kainic acid injection surgery, and the other mice were housed in groups. All mice in the study were randomly assigned to each group. Every effort was made to minimize animal suffering and the number of animals used.

### 2.2 Plasmid Construction

pZac2.1‐GfaABC1D‐EGFP‐Rpl10a was constructed from pZac2.1‐GfaABC1D‐hM3D‐mCherry‐SV40 (#92284; Addgene). pZac2.1‐GfaABC1D‐hM3D‐mCherry‐SV40 was processed with NheI (LT‐10‐SC‐622; LABTAS+) and XbaI (LT‐10‐SC‐634; LABTAS+) to remove hM3D‐mCherry. The PCR‐amplified EGFP‐fused mouse Rpl10a sequence (provided by Dr. Morikawa) was then cloned under the GfaABC1D promoter using In‐Fusion Snap Assembly Master Mix (638,947; TaKaRa Bio Inc.). Similarly, pAAV‐GfaABC1D‐SaCas9‐U6‐sgControl and pAAV‐GfaABC1D‐SaCas9‐U6‐sgIgfbp2 were constructed from pAAV‐FLEX‐SaCas9‐U6‐sgRNA (#124844; Addgene) by combining PCR‐amplified GfaABC1D promoter, SaCas9, SV40 polyA, U6‐sgRNA. The sgRNAs targeting Igfbp2 were designed as previously described (Hunker et al. 2020). The possible sgRNAs were determined by uploading the sequence of the most upstream common coding exon to CHOPCHOP (crispor.tefor.net). The selected sgRNAs for Igfbp2 are as follows: sgIgfbp2 #1: 5′‐GGTTCTCCACCAGGCCTCCCT‐3′, sgIgfbp2 #2: 5′‐GGAGGCCTGGTGGAGAACCAC‐3′.

### 2.3 Adeno‐Associated Virus Production

Recombinant AAV was generated as described previously (Morikawa et al. 2021). Briefly, the 293 AAV cell line (AAV‐100; Cell Biolabs Inc.) with AAVdj rep‐cap, pHelper from the AAV‐DJ Helper Free Packaging System (VPK‐400‐DJ; Cell Biolabs Inc.) and transfer plasmids (pZac2.1‐GfaABC1D‐EGFP‐Rpl10a or pAAV‐GfaABC1D‐SaCas9‐U6‐sgControl, pAAV‐GfaABC1D‐SaCas9‐U6‐sgIgfbp2 #1, #2) were used with PEI‐Max (24765; Polysciences Inc.). AAV vectors were purified using the AAVpro Purification Kit All Serotypes (6666; Takara Bio Inc.). Virus titers were determined by qPCR using the AAV2 ITR primer pair, Luna Universal qPCR Master Mix (M3003S; New England Biolabs), or THUNDERBIRD Next SYBR qPCR Mix (QPX‐201; TOYOBO) and the LightCycler qPCR 2.0 system (DX400; Roche).

### 2.4 Stereotaxic Surgery for Virus Infection

Six‐week‐old male C57BL/6J mice were used. Mice were anesthetized with 3%–4% isoflurane. Anesthesia was maintained with 1.5% isoflurane. Mice were then placed in a stereotaxic apparatus and 1 μL of virus was injected into the right CA3 region (AP: −1.8 mm, ML: +2.1 mm, DV: −2.0 mm) at a rate of 200 nL/min using a glass pipette. The virus was allowed to spread from the tract by holding the glass pipette in place for 10 min. The glass pipette was then slowly withdrawn. The titers of the injected AAVs were as follows: AAV_DJ_‐GfaABC1D‐EGFP‐Rpl10a (1.9 × 10^12^ viral genomes (vg/mL)), AAV_DJ_‐GfaABC1D‐SaCas9‐U6‐sgControl (2.9 × 10^11^–1.45 × 10^12^ vg/mL), AAV_DJ_‐GfaABC1D‐SaCas9‐U6‐sgIgfbp2 mix (sgIgfbp2 #1 + #2, 2.15 × 10^11^ vg/mL for each AAV, the total titer was 4.3 × 10^11^ vg/mL). The sgIgfbp2 mix comprised sgIgfbp2#1 and sgIgfbp2#2, and the titer of each AAV was 2.15 × 10^11^ vg/mL.

### 2.5 Intrahippocampal Injection of Kainic Acid

To generate MTLE model mice, six‐week‐old male C57BL/6J mice or 6–8‐week‐old male and female Aldh1l1‐EGFP mice were intrahippocampally injected with kainic acid as previously described (Bielefeld et al. 2017; Araki et al. 2020). Briefly, mice were anesthetized with isoflurane and then placed in a stereotaxic apparatus. 50 nL of 20 mM kainic acid (0222, Tocris) was injected into the right dentate gyrus (AP: −2.0 mm, ML: +1.5 mm, DV: −2.0 mm) at a rate of 25 nL/min using a glass pipette. After holding for 2 min, the glass pipette was slowly withdrawn. When mice were injected with AAV, kainic acid injection was performed 2 weeks after AAV injection.

### 2.6 Sample Preparation and Immunohistochemistry

Mice were deeply anesthetized with isoflurane and transcardially perfused with cold phosphate‐buffered saline (PBS) followed by 4% paraformaldehyde (PFA). Brain samples were postfixed with 4% PFA overnight at 4°C. Coronal hippocampal sections (100 μm) were prepared using a vibratome (Dosaka EM). Coronal hippocampal sections (50 μm) were prepared using a cryostat (Thermo Fisher Scientific) at −20°C after immersion in 30% sucrose/PBS at 4°C for 24 to 48 h.

The slices were then permeabilized and blocked for 1 h at room temperature in 0.1 M PBS containing 0.3% Triton X‐100 and 10% goat serum. The samples were then incubated with primary antibodies in 0.1 M PBS with 0.3% Triton X‐100 and 10% goat serum overnight at 4°C. For pSMAD staining, samples were incubated with primary antibodies for 3 overnights at 4°C. After three rinses with 0.1 M PBS, the samples were incubated with secondary antibodies in 0.1 M PBS with 0.3% Triton X‐100 and 10% goat serum overnight at 4°C. Finally, samples were rinsed three times with 0.1 M PBS and embedded in CC/Mount (K002, Diagnostic BioSystems) or PermaFluor aqueous mounting medium (TA‐030‐FM, Epredia). After the second rinse with PBS, sections were incubated with Hoechst 33342 (1:1000; Invitrogen) for 10 min to visualize nuclei. For Igfbp2 staining, 5% BSA was used instead of 10% goat serum. The primary antibodies used in this study were as follows: guinea pig anti‐NeuN (1:1000; Synaptic Systems), rat anti‐GFAP (1:1000; Invitrogen), chicken anti‐GFP (1:1000; abcam), rabbit anti‐S100β (1:1000; abcam), rabbit anti‐Iba1 (1:500; Wako), rabbit anti‐NeuN (1:500; abcam), goat anti‐Igfbp2 (1:250; R&D Systems), and rabbit anti‐pSMAD (1:250; Cell Signaling Technology). Secondary antibodies conjugated to Alexa Fluor dyes (1:500; Invitrogen, 1:500; Jackson Immuno Research) were used.

### 2.7 Fluorescence In Situ Hybridization

Mice were transcardially perfused as mentioned above. Brain samples were postfixed with 4% PFA for at least 2 overnights at 4°C, followed by immersion in 30% sucrose in PBS at 4°C overnight. Prepared coronal hippocampal slices (50 μm) using a cryostat at −20°C, were stored in 4% PFA at 4°C for at least 2 overnights. Sections were washed twice in 0.1 M PB and twice in 0.75% glycine/0.1 M PB, then permeabilized in 0.3% Triton X‐100/0.1 M PB for 20 min at room temperature. After washing in 0.1 M PB, the samples were incubated in 2 mg/mL Proteinase K (Wako) at 37°C for 30 min. The samples were then acetylated for 10 min in acetylation solution containing acetic anhydride. After washing with 0.1 M PB, the sections were incubated in hybridization solution at 65°C for 1 h. The hybridization solution consisted of 20× SSC; 12.5 mL, 10% blocking reagent; 10 mL, formamide; 25 mL, 2% N‐lauroylsarcosine (NLS); 2.5 mL, 10% SDS; 0.5 mL. Sections were transferred to a new hybridization solution containing 500 ng RNA probe for *Igfbp2* and allowed to react overnight at 65°C. The sections were then washed twice at 65°C in 2× SSC/50% formamide/0.1% NLS and incubated in 20 μg/mL RNase A (Nippon Gene) in RNase solution (10 mM Tris–HCl [pH 7.5]/1 mM EDTA/0.5 M NaCl) for 30 min at 37°C. After washing twice with 2× SSC/0.1% NLS, twice with 0.2% SSC/0.1% NLS at 37°C and in Tris solution at pH 7.5 (TS7.5; 0.1 M Tris–HCl [pH 7.5]/0.15 M NaCl), the samples were blocked for 1 h in 1% blocking reagent in TS7.5. The sections were then incubated with primary antibodies in 1% blocking reagent solution for 2 h at room temperature. After three washes in TS7.5 containing 0.05% Tween20 (TNT), the sections were incubated with secondary antibodies in 1% blocking reagent solution for 90 min at room temperature. After three washes with TNT, the sections were equilibrated in a Tris solution at pH 8.0 (TS8.0; 0.1 M Tris–HCl [pH 8.0]/0.1 M NaCl/10 mM MgCl_2_). The sections were incubated with SIGMAFAST Fast Red TR/Naphthol AS‐MX Tablets (Sigma Aldrich) for 40 min at room temperature. The sections were washed three times with PBS containing 10 mM EDTA and mounted with CC/Mount. During the second wash with PBS, 0.1% Hoechst 33342 was added. The primary antibodies used in this study were Anti‐digoxigenin‐AP, Fab fragments (1:1000, Roche), and rabbit anti‐S100β (1:1000, abcam). Secondary antibodies used were: Alexa Fluor 488‐conjugated secondary antibodies (1:500, Thermo Fisher Scientific). nt474‐1206 from NM_008342.2 was used as an RNA probe for *Igfbp2*.

### 2.8 RNA Extraction From Hippocampal Astrocytes

Astrocytic RNA was extracted from the hippocampus of mice injected with AAV_DJ_‐GfaABC1D‐EGFP‐Rpl10a. The right hippocampus was dissected and collected from three to four mice per sample. The collected hippocampus was homogenized with 500 mL of cold homogenization buffer (50 mM Tris‐pH 7.5, 12 mM MgCl_2_, 1% NP‐40, 100 μg/mL cycloheximide, 100 mM KCl, 1× HALT protease inhibitor, 0.5 mM DTT, 1 mg/mL sodium heparin, 0.2 units/mL RNasin). The homogenate was centrifuged and the supernatant was collected. 5%–10% of the collected supernatant was aliquoted as an input sample containing RNA from the entire hippocampus. The remaining supernatant was incubated and rotated with 2 mL of rabbit anti‐GFP antibody (ab290; abcam) at 4°C for 4 h. Then 100 mL of magnetic beads (1004D; Invitrogen) were added and rotated overnight at 4°C. On the magnetic stand, the sample was washed three times with high‐salt buffer (50 mM Tris‐pH 7.5, 12 mM MgCl_2_, 1% NP‐40, 100 mg/mL cycloheximide, 0.5 mM DTT, 300 mM KCl). The remaining immunoprecipitated sample was the IP sample containing RNA enriched in GFP‐tagged ribosome‐expressing astrocytes. RNA was extracted from the input and IP samples using the RNeasy plus mini kit (QIAGEN).

### 2.9 RNAseq Analysis

Multiplexed libraries were prepared from RNA extracted from input and IP samples using the NEBNext Ultra II RNA Library Prep Kit for Illumina (E7770; New England Biolabs). Sequencing was performed on Illumina NovaSeq 6000 (Illumina). In this study, RNAseq was performed in triplicate for the AAV control group (#1–3) in which only AAV was injected, the kainic acid group (#1–3) in which kainic acid was administered after AAV injection, and the PBS group (#1–3) in which PBS was administered after AAV injection. In the first sequence, AAV control group #1, 2 and kainic acid group #1, 2 were sequenced; in the second sequence, AAV control group #3 and kainic acid group #3 were sequenced; in the third sequence, PBS group #1, 2, and 3 were sequenced. To reduce batch effects, samples with RiboTag AAV injection only were treated as the control group. Reads were aligned to the mouse mm10 reference genome using the STAR spliced read aligner. Differential gene expression analysis was performed on genes with Fragments per kilobase of exon per million mapped reads (FPKM) > 1 in all comparison samples using the Bioconductor packages edgeR and limmaVoom with a false discovery rate (FDR) threshold < 0.05 (http://www.bioconductor.org/). Genes with Log2‐fold change ≥ 1 or ≤ −1 and adjusted *p* value < 0.05 were designated as differentially expressed genes (DEGs). DEGs that were more than 2‐fold higher in the IP samples than in the input samples were designated as astrocyte‐enriched DEGs (Yu et al. 2020). Gene ontology enrichment analysis was performed using ShinyGO (http://bioinformatics.sdstate.edu/go/). VolcanoseR (https://goedhart.shinyapps.io/VolcaNoseR/) was used to plot each gene based on log2 fold change and ‐log10 adjusted *p* value.

### 2.10 Electrode Implantation

The electrode assembly consisted of 5 electrodes for local field potentials (LFPs) and 2 stainless steel screw electrodes for ground/reference. Nichrome wire was used for the LFP electrodes, and the tips of the electrodes were plated with platinum to lower electrode impedances to 150–300 kΩ at 1 kHz. Electrode implantation was performed at least 14 days after kainic acid administration. After isoflurane anesthesia, mice were placed in a stereotaxic apparatus. The tips of the LFP electrodes were then placed on the brain surface (AP: −2.0 mm, ML: +1.5 mm) and lowered 2.0 mm from the surface. Two screw electrodes were also implanted on the cerebellar surface as ground/reference electrodes. All electrode sets were fixed to the skull with dental cement. The location of electrodes was confirmed histologically after cresyl violet staining. Data from electrodes with the electrode tip located in the hippocampal dentate gyrus were used for analysis.

### 2.11 Video and LFP Monitoring

After a recovery period of at least 5 days from the surgery, LFP recordings were performed (Yoshimoto et al. 2021). On the day before recording, the mice were allowed to explore the recording cage for 15 min for habituation. The electrode assembly was connected to the Cereplex M digital headstage (Blackrock Neurotech) and the digital signals were transferred to the CerePlex Direct (Blackrock Neurotech). LFP data were sampled at 2 kHz and lowpass‐filtered at 500 Hz. LFP data were recorded for a period of 4 × 1 h sessions (Sierra et al. 2015). Mouse behavior was recorded using a web camera placed above the recording apparatus during the experiment.

### 2.12 The Analysis of Spontaneous Recurrent Seizures (SRSs)

SRSs were detected using MATLAB custom routines as previously described (Gu et al. 2015; Lentini et al. 2021; Lisgaras and Scharfman 2022). Briefly, the neural signal was high‐pass filtered at 5 Hz. The baseline of the high‐pass filtered LFPs was manually determined by skilled experimenters, and the SD of the baseline signals was calculated. The high‐pass filtered LFPs with an amplitude of 3 × SD of the baseline that lasted for more than 10 s were defined as an SRS.

### 2.13 In Vitro Electrophysiology

Electrophysiological recordings from dentate granule cells were performed as described previously with some modifications (Nakashima et al. 2022). Acute hippocampal slices were prepared from 3‐ to 4‐week‐old mice. Mice were decapitated under isoflurane anesthesia, and the brain was quickly removed. The brain was then submerged in ice‐cold oxygenated (95% O_2_/5% CO_2_) modified artificial cerebrospinal fluid (mACSF) containing 27 mM NaHCO_3_, 1.5 mM NaH_2_PO_4_, 2.5 mM KCl, 0.5 mM ascorbic acid, 1 mM CaCl_2_, 7 mM MgSO_4_ and 222.1 mM sucrose. Horizontal brain blocks containing the hippocampus and entorhinal cortex were sliced at a thickness of 300 μm at a speed of 0.1 mm/s using a Leica vibratome (VT1200S, Leica Microsystems). After the slices were transferred to oxygenated artificial cerebrospinal fluid (ACSF) containing 127 mM NaCl, 26 mM NaHCO_3_, 1.6 mM KCl, 1.24 mM KH_2_PO_4_, 1.3 mM MgSO_4_, 2.4 mM CaCl_2_, and 10 mM glucose, the slices were treated with 10 ng/mL of recombinant mouse Igfbp2 (#797‐B2‐025, R&D systems) for at least 1 h at room temperature. Recombinant mouse Igfbp2 was dissolved in ultrapure water at a concentration of 100 μg/mL as a stock and diluted 1:10000 with ACSF. Experiments were performed in a submerged chamber perfused at 3–4 mL/min with oxygenated ACSF containing 10 ng/mL of recombinant Igfbp2 at 32°C. Borosilicate glass pipettes (4–8 MΩ) were filled with a potassium‐based solution containing 120 mM potassium gluconate, 5 mM KCl, 10 mM HEPES, 10 mM disodium phosphocreatine, 2 mM Mg‐ATP, 0.1 mM Na2‐GTP, 0.2 mM EGTA, and 0.2% biocytin. Slices were visualized using an upright microscope (ECLIPSE‐FN1; Nikon) fitted with an infrared differential interference contrast, 40× water immersion objective (CFI Apo NIR 40XW; Nikon), halogen lamp (LS‐DWL‐N; Sumita optical glass) and fluorescent lamp (C‐LHG1; Nikon). Neurons were imaged with a CCD camera (C3077‐78; Hamamatsu Photonics). The signals were amplified and digitized at a sampling rate of 20 kHz using a MultiClamp 700B amplifier and a Digidata 1440A digitizer controlled by Clampex 10.7 software (Molecular Devices). The liquid junction potentials were automatically corrected before each experiment using Pipette Offset mode (Molecular Devices) and were not corrected post hoc. The dentate granule cells were injected with a step current injection for 800 ms (0 pA to 200 pA, 20 pA steps) to determine the intrinsic action potential properties. After recording, the slices were fixed in 4% PFA/PB at 4°C overnight. To visualize the recorded neurons, the slices were permeabilized in 5% BSA and 0.3% Triton X‐100 in PBS (5% BSA/PBST) for 1 h at room temperature. Subsequently, the slices were incubated overnight with streptavidin‐conjugated Alexa Fluor 594 solution at a concentration of 1 μg/mL in 5% BSA/PBST. The labeled slices were costained with DAPI (1:2000; D9542–10MG, Sigma Aldrich) in 0.1 M PBS for 10 min at RT and mounted onto microscope slides with VECTASHIELD Vibrance Antifade Mounting Medium (H‐1700; Vector Laboratories Inc.).

### 2.14 Image Acquisition

The immunostained samples were analyzed using a FV1200 (Olympus) or Nikon A1‐HD25 (Nikon) confocal system under 10×, 20×, and 40× objectives. Cresyl violet‐stained samples were analyzed with a BZ‐X710 (Keyence) phase‐contrast microscope to confirm the electrode position. ImageJ (NIH, Bethesda, MD) was used for analysis.

### 2.15 Quantification

Z‐series images were collected in 1‐μm steps, and 11Z sections (10 μm thick) were stacked using ImageJ to quantify the GFAP‐positive area, colocalization between Igfbp2 and cell‐type‐specific markers, and hippocampal Igfbp2 expression levels. Twenty‐one Z‐series sections (0.5‐μm steps, 10 μm thick) were collected for quantification of colocalization between EGFP‐Rpl10a and S100β and for quantification of astrocytic pCREB expression levels.

Quantification of GFAP‐positive area or Igfbp2‐positive area was performed as follows: on the Z‐stacked images (GFAP) or the Z‐stacked tiled images (Igfbp2), GFAP or Igfbp2 immunofluorescence signals were detected by the threshold of fluorescence intensities in ImageJ. Then, the percentage of positive area in the total area of the region of interest (ROI) was calculated.

Quantification of colocalization between Igfbp2 and cell type‐specific markers (S100β, Iba1, and NeuN) was performed as follows: on each section, Igfbp2, S100β, Iba1, and NeuN immunofluorescence signals were detected by determining the threshold of fluorescence intensities in ImageJ. Colocalized images of Igfbp2 and S100β, Iba1, or NeuN were then prepared for each section. The colocalization ratio was calculated by dividing the area of Z‐stacked colocalized images by the area of Z‐stacked Igfbp2 images.

Quantification of colocalization between EGFP‐Rpl10a and S100β was performed by counting the number of cells on the Z‐stacked images using the Cell Counter plugin. We divided the number of colocalized cells by the number of S100β‐positive cells.

Quantification of astrocytic pCREB expression levels was performed as follows: on the stacked images, we selected the ROI containing the astrocytic soma and measured the pCREB immunofluorescence intensity in the ROI. The average fluorescence intensity of five background ROIs (selected from regions without nuclei) was subtracted from the measured pCREB intensity. If the subtracted value was less than 0, the value was replaced with 0. The resulting value was used for quantification.

### 2.16 Experimental Design and Statistical Analysis

No statistical methods were used to pre‐determine the sample size, but our sample sizes are similar to those reported in previous publications (Lentini et al. 2021; Sierra et al. 2015; Zhou et al. 2019). Data were collected and statistically analyzed in a blinded fashion to avoid bias. Sigma‐Plot 12.0, RStudio and MATLAB2022a software were used to analyze the data. Data are presented as the mean ± standard error of the mean. Student's *t*‐test, Mann–Whitney rank sum test, one‐way analysis of variance (ANOVA) followed by Tukey's test, extra sum‐of‐squares F test, Kolmogorov–Smirnov test, or bootstrap resampling test were used for statistical analysis.

In the bootstrap resampling test, the resampled data were obtained from the mixed data of the two groups and the difference in means was calculated. This procedure was repeated 10,000 times. The null hypothesis was rejected if the two‐sided 95% confidence interval for the difference in means obtained by resampling did not contain the true difference in means.

### 2.17 Code Availability

All codes used in this study are available from the corresponding authors upon reasonable request.

### 2.18 RNA‐seq data deposition

RNA‐seq data have been deposited in the Gene Expression Omnibus (GEO) repository (https://www.ncbi.nlm.nih.gov/geo) with accession numbers (GSE237321). Lead contact will fulfill reasonable requests for other data.

## 3 Result

### 3.1 Astrocyte‐Focused Transcriptional Profiles in the Epileptic Hippocampus

To establish a mouse model of MTLE with hippocampal sclerosis, we injected kainic acid (KA), a glutamate receptor agonist, into the right dentate gyrus of the hippocampus (Bielefeld et al. 2017). This intrahippocampal KA‐injection model recapitulates multiple pathological features of hippocampal sclerosis, including reactive astrogliosis, microgliosis, extensive pyramidal cell loss, and granule cell dispersion (Sierra et al. 2015; Araki et al. 2020). In addition, the model exhibits resistance to certain anti‐seizure medications, and its use in novel drug discovery is endorsed by the Epilepsy Therapy Screening Program (Kehne et al. 2017; Löscher et al. 2020).

**FIGURE 1:**
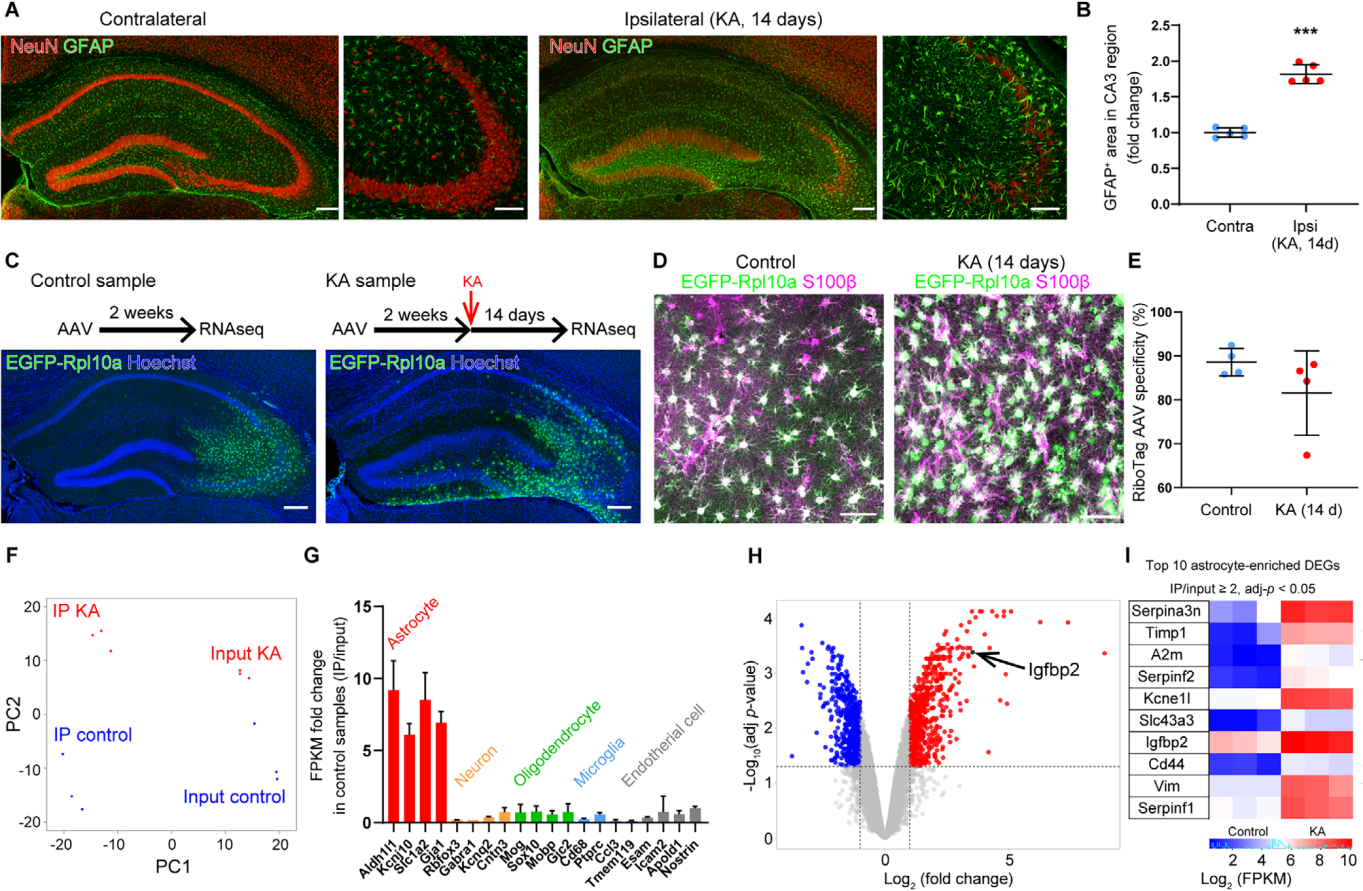
Astrocyte‐focused transcriptional profiling reveals altered gene expression in the epileptic hippocampus. (A) Representative confocal images of the hippocampus from KA‐injected MTLE model mice. Scale bars, 200 μm (left), 100 μm (right, magnified image). (B) Quantification of GFAP^+^ area in the CA3 region. *n* = 5 mice (2 slices per mouse). Data represent mean ± SEM. ***p < 0.001, Student's *t*‐test. (C) Experimental timeline and representative images illustrating astrocyte‐specific RNA‐seq of the CA3 region using AAV‐RiboTag and TRAP. In the control group, RNA was collected 2 weeks after AAV injection. In the KA group, KA was injected 2 weeks after AAV injection, and RNA was collected 14 days post‐KA. Lower panels show EGFP‐Rpl10a expression in the CA3 region. Scale bar, 200 μm. (D) Representative confocal images showing EGFP‐Rpl10a and S100β expression in the CA3 region. Scale bar, 50 μm. (E) Quantification of EGFP‐Rpl10a^+^ cells co‐expressing S100β. Approximately 90% of GFP^+^ cells were S100β^+^ in both groups. *n* = 4 mice (2–3 slices per mouse), mean ± SEM. (F) Principal component analysis (PCA) of CPM data. (G) Expression ratios of cell‐type‐specific markers (FPKM) comparing immunoprecipitated (IP) vs. input samples. *n* = 3 samples per group. (H) Volcano plot showing log_2_ fold changes and –log_10_ adjusted *p* values for astrocyte‐expressed genes (FPKM > 1 in all samples). Differentially expressed genes (DEGs) were defined by adjusted *p* < 0.05 and > 2‐fold change. (I) Heatmaps of the top 10 upregulated or downregulated astrocyte‐enriched DEGs (IP/input ≥ 2), shown as FPKM values.

To validate the presence of reactive astrogliosis, we performed immunohistochemical analysis 14 days after KA injection. In the CA3 region of the hippocampus—an area well known for reactive astrogliosis in both MTLE patients and rodent models—we observed NeuN‐positive pyramidal cell loss and a significant increase in the area labeled with the astrocytic marker GFAP in the ipsilateral hippocampus (Figure 1A,B, *p* = 0.00000178, *t*_(8)_ = −12.3, Student's *t*‐test).

To examine transcriptomic changes associated with reactive astrogliosis, we combined AAV injection into the CA3 region with Translating Ribosome Affinity Purification (TRAP) to isolate mRNA specifically from astrocytes in that region (Figure 1C) (Yu et al. 2018; Yu et al. 2020; Bravo‐Ferrer et al. 2022). TRAP allows immunoprecipitation of ribosome‐bound mRNA and offers several advantages over conventional methods, such as fewer artifacts compared to fluorescence‐activated cell sorting (FACS), and the ability to capture mRNAs being actively translated, thus better reflecting protein expression profiles (Haimon et al. 2018; Yu et al. 2020).

Leveraging these features, we compared gene expression profiles between reactive astrocytes in the CA3 region and other hippocampal cells, aiming to identify astrocyte‐specific transcriptional signatures. To this end, we used RiboTag AAV (AAV_DJ_‐GfaABC1D‐EGFP‐Rpl10a), which expresses a GFP‐tagged ribosomal protein (EGFP‐Rpl10a) under the control of an astrocyte‐specific promoter, to selectively label ribosomes in CA3 astrocytes (Figure 1C). We then performed immunoprecipitation with an anti‐GFP antibody to purify translational mRNA from astrocytes in the CA3 region.

For transcriptomic comparison, we prepared two groups: a control group, in which astrocytic mRNA was collected from hippocampi injected with RiboTag AAV alone, and a KA group, in which astrocytic mRNA was isolated from hippocampi injected with KA following RiboTag AAV administration (Figure 1C).

We confirmed that approximately 90% of GFP‐positive cells in the CA3 region co‐expressed the astrocytic marker S100β (Figure 1D,E). We then performed RNA sequencing on immunoprecipitated (IP) samples enriched for astrocytic mRNA, as well as on input samples representing total hippocampal RNA. Principal component analysis (PCA) based on counts per million (CPM) revealed clear distinctions between control and KA samples, as well as between IP and input samples (Figure 1F). Analysis of cell‐type–specific marker genes further showed that astrocytic markers were enriched, while markers of other cell types were depleted, in the IP samples relative to the input samples (Figure 1G). These results confirm the suitability of our approach for investigating transcriptional changes in hippocampal astrocytes.

To identify differentially expressed genes (DEGs) induced by KA injection, we compared genes with fragments per kilobase of transcript per million mapped reads (FPKM) > 1 in all immunoprecipitated (IP) samples between the control and KA groups, using a false discovery rate (FDR) < 0.05. Genes were considered significantly differentially expressed if they exhibited at least a two‐fold change and an adjusted *p* value < 0.05 (Figure 1H).

TRAP enables the collection of RNA from both astrocytes expressing GFP‐tagged ribosomes (IP) and the total hippocampal RNA (input) from the same mouse. We next identified DEGs enriched in astrocytes by selecting genes with FPKM (IP vs. input) ≥ 2 (Yu et al. 2020). The top 10 astrocyte‐enriched DEGs altered by KA injection are shown in Figure 1I. Several of these genes have been previously associated with epilepsy (Tinnes et al. 2013; Liu et al. 2023). For example, *Serpina3a* is highly expressed in hippocampal astrocytes of epileptic mice, and its knockdown reduces seizure development (Liu et al. 2023). While many of the top 10 genes are linked to inflammatory or reactive astrocyte phenotypes, *Kcne1l* and *Igfbp2* have rarely been associated with these states. Although the role of *Kcne1l* in the brain remains poorly understood, previous studies suggest that *Igfbp2* may influence neuronal excitability (Khan et al. 2019; Shigetomi et al. 2024). Based on this, we prioritized insulin‐like growth factor‐binding protein 2 (Igfbp2) for further investigation among the top candidate astrocyte‐enriched DEGs.

**FIGURE 2:**
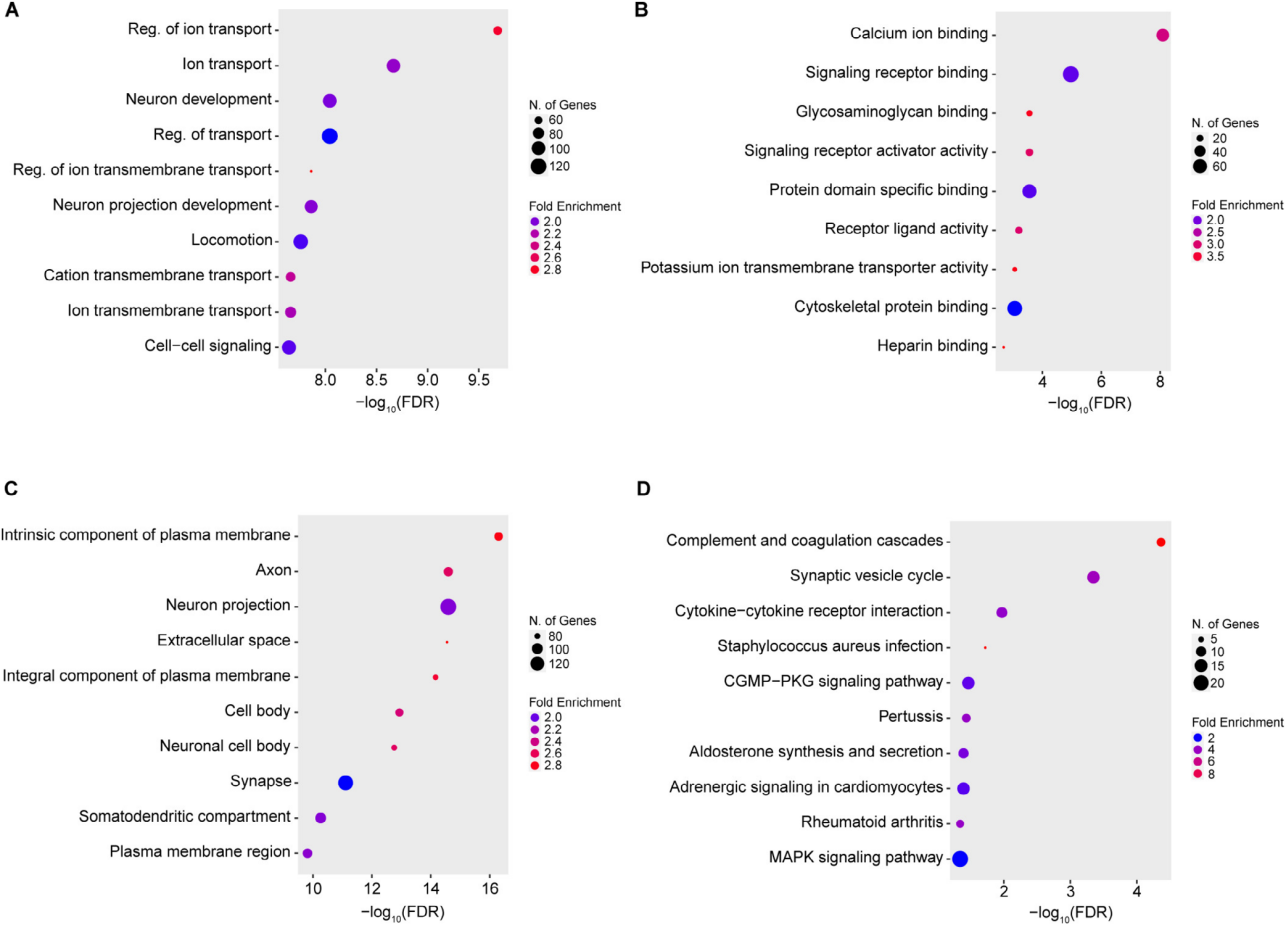
Gene ontology and pathway enrichment analyses of upregulated astrocytic DEGs in the epileptic hippocampus. (A) Gene ontology (GO) enrichment analysis of biological process terms associated with upregulated DEGs (adjusted *p* < 0.05, log_2_ fold change ≥ 1). (B) GO enrichment analysis of molecular function terms for the same DEG set. (C) GO enrichment analysis of cellular component terms for upregulated DEGs. (D) Kyoto encyclopedia of genes and genomes (KEGG) pathway enrichment analysis of upregulated DEGs.

Subsequently, we performed gene ontology (GO) enrichment analysis on the upregulated DEGs in the KA group (Figure 2). Within the biological process category, GO terms related to ion transport and neuronal development were significantly enriched (Figure 2A). These findings are consistent with known mechanisms underlying epilepsy, where impaired astrocytic potassium buffering and glutamate transport contribute to hyperexcitability (Rangroo Thrane et al. 2013; Petr et al. 2015; Peterson et al. 2021), and neuronal circuits undergo reorganization during epileptogenesis (Zhou et al. 2019; Lybrand et al. 2021).

In the molecular function category, GO terms associated with potassium ion buffering were also enriched (Figure 2B). In addition, since astrocytes undergo marked morphological changes during the transition to a reactive state, cytoskeleton‐related GO terms were also enriched. Because our focus is on astrocyte and neuron interactions during epileptogenesis, the significant enrichment of genes related to signal transduction is particularly noteworthy. These include genes involved in cell–cell signaling within biological processes, as well as signaling receptor binding, receptor activator activity, and receptor ligand activity within molecular functions (Figure 2A,B).

These results support our hypothesis that reactive astrocytes in the sclerotic hippocampus upregulate genes involved in signal transduction pathways, which may in turn influence neuronal function. Notably, *Igfbp2*, the astrocyte‐enriched DEG we prioritized, falls under the GO term “signal transduction.” Therefore, the GO enrichment analysis further motivated us to explore the effects of Igfbp2 on neuronal excitability.

To mitigate batch effects, we designated the samples derived from hippocampi injected with RiboTag AAV alone as control samples (see the Methods section for detailed sample information). However, it is important to note that these control samples differ from the KA group in certain aspects, including the time course and the invasiveness of the surgical procedure, as KA was administered after AAV injection in the KA group.

To address these potential confounds, we additionally compared the KA group with a phosphate‐buffered saline (PBS) group, in which astrocytic RNA was collected from hippocampi injected with PBS following RiboTag AAV injection. Notably, when comparing the PBS group with the KA group, *Igfbp2* again emerged as one of the top 10 differentially expressed, astrocyte‐enriched genes (Figure S1).

### 3.2 Igfbp2 Is Highly and Specifically Expressed in Astrocytes of the Epileptic Hippocampus

Igfbp2, encoded by the *Igfbp2* gene, has been reported to enhance the firing rate of hippocampal pyramidal neurons in both developing and disease‐mimicking brains, thereby increasing overall brain excitability (Khan et al. 2019; Shigetomi et al. 2024). Recently, Igfbp2 has attracted considerable attention for its role in neuron–astrocyte interactions. Astrocyte‐derived Igfbp2 has been implicated in neural hyperexcitability, the formation of fear‐related memories, and the regulation of neuronal maturation (Caldwell et al. 2022; Sun et al. 2024). Based on these findings, we hypothesized that elevated Igfbp2 expression in astrocytes contributes to increased neuronal excitability and the emergence of SRSs in the MTLE mouse model.

**FIGURE 3:**
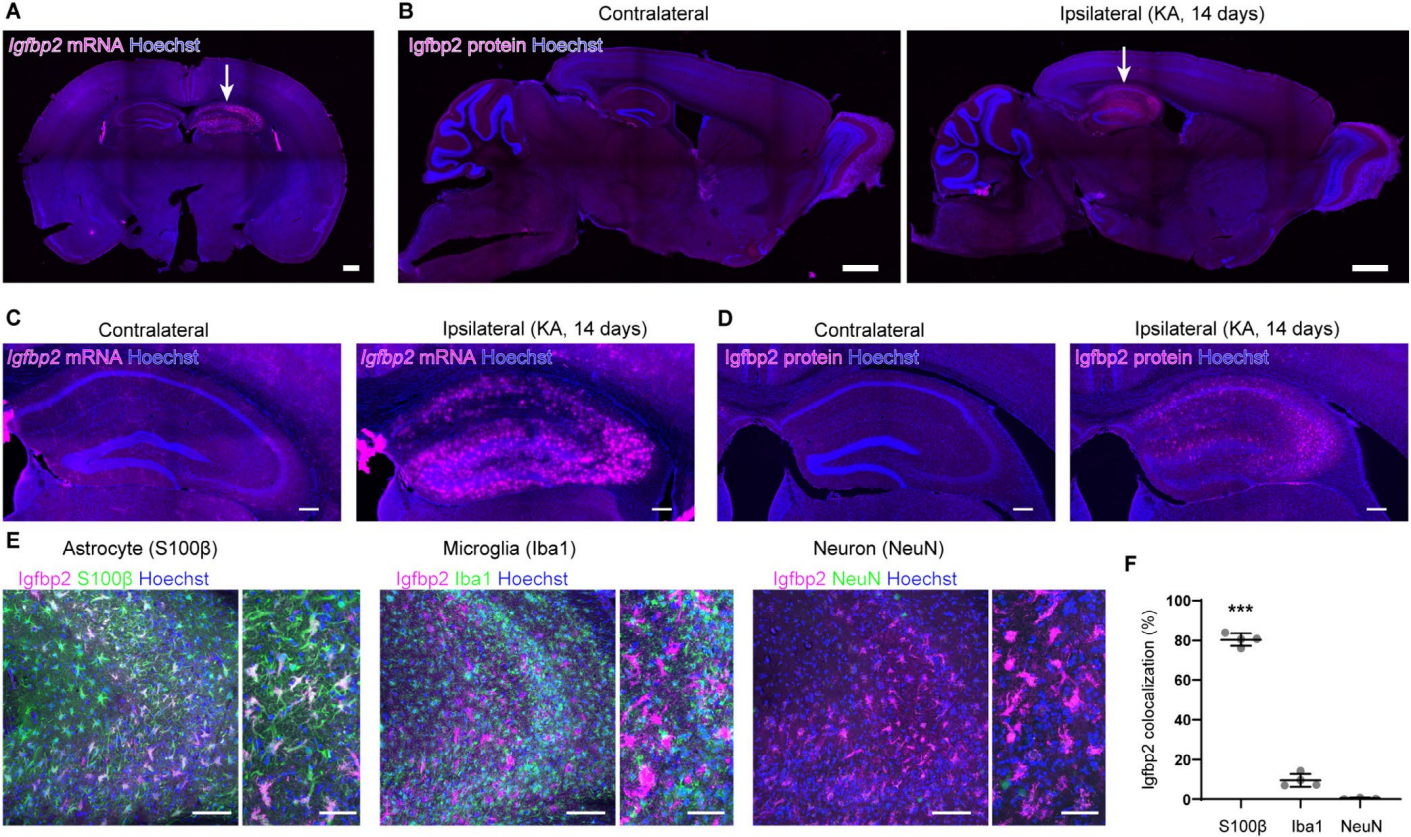
Igfbp2 is selectively upregulated in astrocytes of the epileptic hippocampus. (A) Representative image of *Igfbp2* mRNA expression 14 days after KA injection. The arrow indicates the KA‐injected hippocampus. Scale bar, 500 μm. (B) Representative images of Igfbp2 protein expression in the brain. Igfbp2 expression is restricted to the KA‐injected hippocampus (arrow). Scale bar, 1 mm. (C, D) Representative images showing *Igfbp*2 mRNA (c) and protein (d) expression in the hippocampus of MTLE model mice. Scale bar, 200 μm. (E) Representative images of Igfbp2 co‐expression with S100β (astrocytes), Iba1 (microglia), and NeuN (neurons) in the CA3 region. Scale bars, 100 μm. Insets: Enlarged images with scale bars, 50 μm. (F) Quantification of Igfbp2 colocalization with cell type–specific markers. *n* = 3–4 mice (2 slices per mouse); mean ± SEM. ***p < 0.001, Tukey's test following one‐way ANOVA.

To test this hypothesis, we examined Igfbp2 expression at both the RNA and protein levels in MTLE model mice using in situ hybridization and immunohistochemistry. Strikingly, *Igfbp2* expression was minimal in the contralateral hippocampus and in brain regions outside the hippocampus. In contrast, both Igfbp2 mRNA and protein were significantly upregulated in the hippocampus following KA injection (Figure 3A–D). Notably, this increased expression was predominantly restricted to astrocytes, with minimal detection in microglia or neurons (Figure 3E,F, *F*_(2,8)_ = 882.53, *p* = 4.14 × 10^−10^, one‐way ANOVA, *p* = 2.40 × 10^−9^; S100β vs. Iba1, *p* = 1.74 × 10^−9^; S100β vs. NeuN Tukey's test after ANOVA).

**FIGURE 4:**
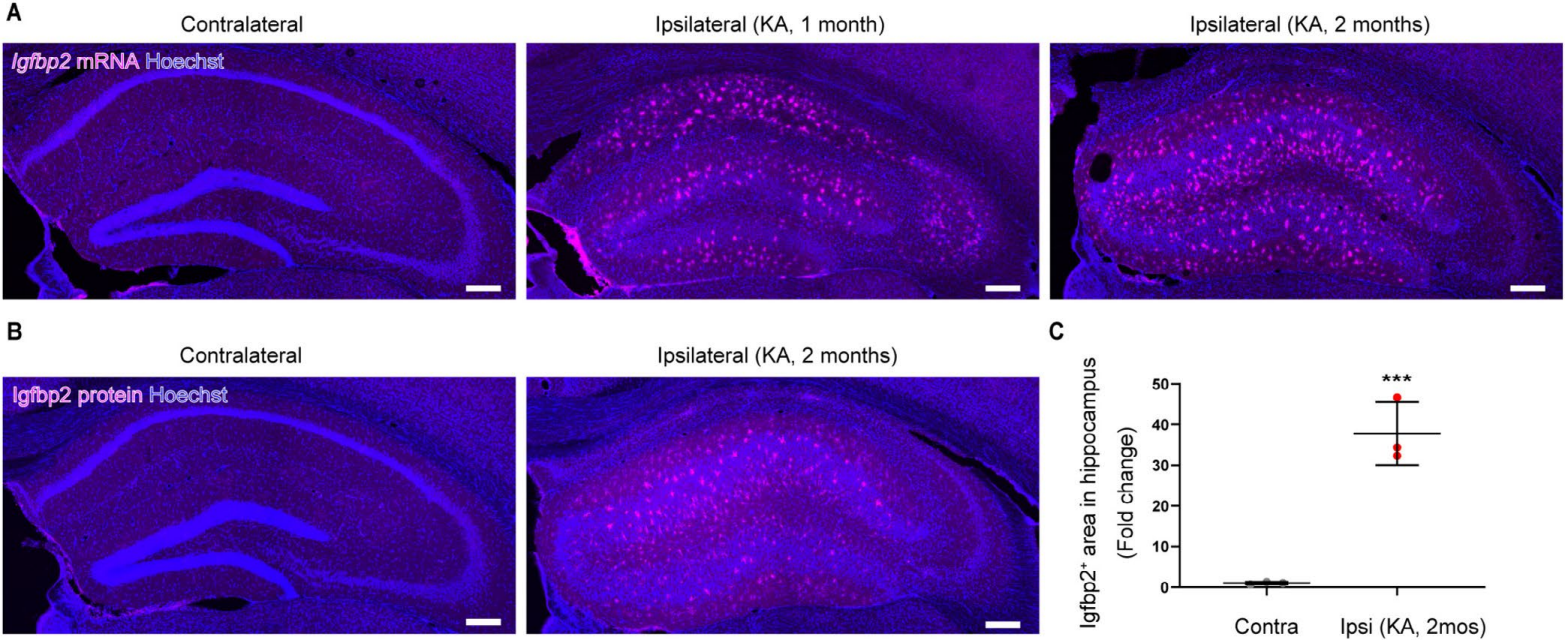
Igfbp2 remains upregulated in the hippocampus 2 months after KA administration. (A) Representative fluorescence in situ hybridization images showing *Igfbp2* mRNA expression at 1 and 2 months after KA administration. Scale bar, 200 μm. (B) Representative images of Igfbp2 protein expression at 1 and 2 months after KA administration. Scale bar, 200 μm. (C) Quantification of Igfbp2 protein expression in the ipsilateral hippocampus 2 months after KA administration. *n* = 3 mice (3 slices per mouse); mean ± SEM. ***p < 0.001, Student's *t*‐test.

Furthermore, the upregulated Igfbp2 expression persisted for at least 2 months after status epilepticus (Figure 4, *p* = 0.0012, *t*_(4)_ = −8.21, Student's *t*‐test), indicating that this upregulation is sustained rather than transient.

Collectively, these findings demonstrate that *Igfbp2* expression is selectively and persistently elevated in astrocytes within the epileptic hippocampus.

Moreover, we performed RNA‐seq analysis on samples collected at 5 days after KA injection to investigate *Igfbp2* expression in astrocytes. Astrocytic *Igfbp2* already showed a tendency toward upregulation at this early time point following KA‐induced status epilepticus (Figure S2). In approximately 40% of mice injected with intrahippocampal KA, initial SRSs typically emerge around one week post‐injection (McGann et al. 2023). Although we confirmed the upregulation of Igfbp2 two months after status epilepticus, SRSs remain detectable at this time point in MTLE model mice (Lentini et al. 2021). These findings suggest that the temporal dynamics of Igfbp2 expression during epileptogenesis may be associated with the onset and persistence of SRSs.

### 3.3 Astrocytic Igfbp2 Contributes to the Development of SRSs

**FIGURE 5:**
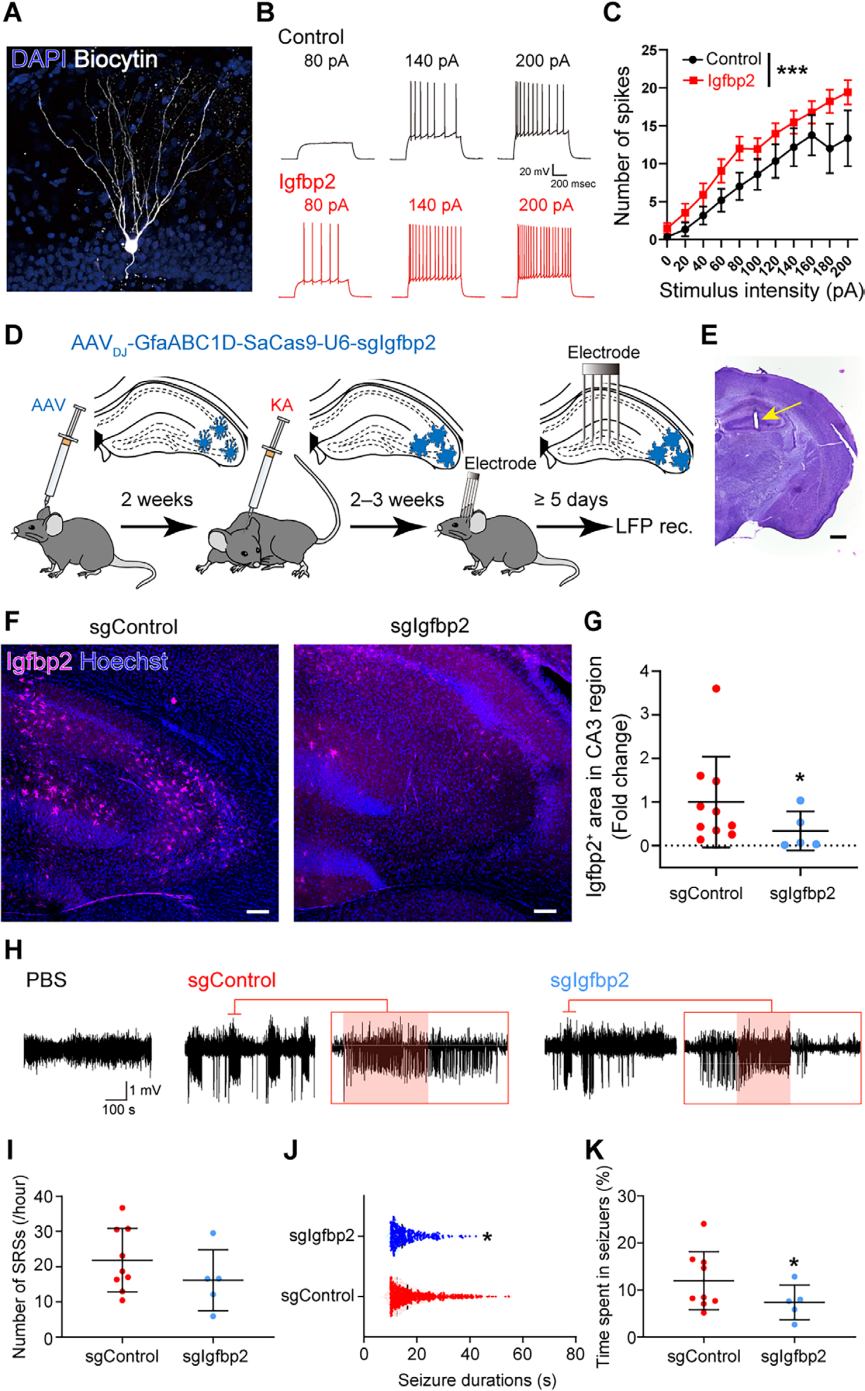
Astrocytic Igfbp2 deletion ameliorates the development of spontaneous recurrent seizures (SRSs). (A) Representative image of a biocytin‐filled dentate granule cell used for electrophysiological recordings. (B) Representative whole‐cell current‐clamp recording from a dentate granule cell. (C) Stimulus–response curves of dentate granule cells in the presence or absence of Igfbp2. *n* = 12 (ACSF) and 19 (Igfbp2) cells; mean ± SEM. ***p < 0.001, extra sum‐of‐squares *F* test. (D) Experimental timeline for assessing the effects of astrocyte‐specific Igfbp2 deletion on SRS development. AAVs encoding SaCas9 and sgRNAs targeting *Igfbp2* were injected into the hippocampus. Two weeks later, KA was administered intrahippocampally. After another 2 weeks, LFP electrodes were implanted into the dentate gyrus, followed by recovery and recording. (E) Histological verification of electrode placement (arrow). (F) Representative images showing Igfbp2 expression in the hippocampus following AAV delivery of sgControl or sgIgfbp2. Scale bar, 100 μm. (G) Quantification of Igfbp2 expression in the CA3 region. *n* = 10 mice (sgControl) and 5 mice (sgIgfbp2); 2 slices per mouse; mean ± SEM. *p < 0.05, bootstrap resampling test. (H) Representative LFP traces recorded from the dentate gyrus. Red bars indicate automatically detected SRS events. Left: PBS‐injected mouse; middle: sgControl‐expressing MTLE mouse; right: SgIgfbp2‐expressing MTLE mouse. Sustained, high‐amplitude rhythmic activity characteristic of SRSs was observed in MTLE model mice. (I) Quantification of SRS frequency. A trend toward reduced SRS frequency was observed in sgIgfbp2‐expressing mice. *n* = 9 (sgControl) and 5 (sgIgfbp2) mice; mean ± SEM. (J) Quantification of SRS duration. *n* = 778 events (sgControl) and 326 events (sgIgfbp2); mean ± SEM. *p < 0.05, Kolmogorov–Smirnov test. (K) Quantification of cumulative SRS duration during the recording period. *n* = 9 (sgControl) and 5 (sgIgfbp2) mice; mean ± SEM. *p < 0.05, bootstrap resampling test.

Given the elevated expression of Igfbp2 in astrocytes within the sclerotic hippocampus of epileptic mice, we investigated whether Igfbp2 contributes to the increased excitability of dentate granule cells. To address this, we prepared acute hippocampal slices from naïve mice and performed whole‐cell patch‐clamp recordings from dentate granule cells. Notably, treatment with 10 ng/mL of Igfbp2 significantly enhanced granule cell excitability, as demonstrated by an increased number of action potentials in response to current injection (Figure 5A–C, *F*_(DFn,DFd)_ = 45.37_(4,14)_, *p* < 0.001, extra sum‐of‐squares F test).

Subsequently, we sought to assess the impact of astrocytic Igfbp2 expression on SRSs in MTLE model mice. To this end, we utilized the CRISPR/SaCas9 system to induce astrocyte‐specific mutagenesis of *Igfbp2*, employing *Staphylococcus aureus* Cas9 (SaCas9) and a single guide RNA (sgRNA) targeting *Igfbp2* (Figure 5D). The sgRNAs were designed to target exon 2, which is the most 5′ located coding exon shared by all *Igfbp2* transcript variants (Hunker et al. 2020).

Increased neurogenesis in the dentate gyrus (DG) following status epilepticus is known to contribute to epileptogenesis (Cho et al. 2015; Luo et al. 2016). However, recent studies have shown that AAV delivery to the DG can impair adult neurogenesis (Johnston et al. 2021). To minimize potential adverse effects of AAV injection into the DG, we instead targeted the CA3 region for AAV delivery, consistent with the approach used for RiboTag AAV injection.

AAV‐mediated delivery of SaCas9 using AAV_DJ_‐GfaABC1D‐SaCas9‐pU6‐sgIgfbp2 significantly reduced Igfbp2 expression in the CA3 region of KA‐injected mice (Figure 5F,G, *p* < 0.05, bootstrap resampling test).

To SRSs, we recorded LFPs within the ipsilateral hippocampal dentate gyrus. The AAV carrying SaCas9 was delivered at least 2 weeks before the intrahippocampal administration of KA. Since mice in the MTLE model, generated by intrahippocampal KA injection, typically develop epileptogenesis and begin exhibiting SRSs within 2 weeks of injection (Lisgaras and Scharfman 2022), we implanted electrodes no earlier than 2 weeks after KA administration. LFPs were then recorded at least 5 days after electrode implantation (Figure 5D,E).

Comparison between the MTLE model mice injected with PBS into the hippocampus and the MTLE model mice in which Igfbp2 was deleted specifically in astrocytes revealed distinct characteristics. The MTLE model mice exhibited SRSs as hippocampal paroxysmal discharges, characterized by sustained rhythmic high‐amplitude activity in the LFPs (Figure 5H). Following previous studies, we defined SRSs as LFP events with a frequency greater than 5 Hz and an amplitude exceeding three times the standard deviation of the baseline, lasting for more than 10 s (Gu et al. 2015; Lentini et al. 2021; Lisgaras and Scharfman 2022).

Using this definition, we analyzed the SRSs recorded in the LFPs and quantified various seizure parameters, including seizure frequency, seizure duration, and cumulative seizure duration within the recording period (i.e., time spent in seizures). Control AAV‐injected MTLE model mice exhibited an average SRS frequency of approximately 20 events per hour (Figure 5I), consistent with previous reports (Zeidler et al. 2018; Lentini et al. 2021). Deletion of Igfbp2 in astrocytes did not significantly reduce the frequency of SRSs (Figure 5I).

Notably, the astrocytic Igfbp2‐deleted MTLE model mice showed shorter seizure duration and spent significantly less time in SRSs compared to the control group (Figure 5J, *D* = 0.15, *p* = 0.000040, Kolmogorov–Smirnov test, Figure 5K, *p* < 0.05, bootstrap resampling test). The degree of suppression in seizure duration observed in this study, approximately 50%, was comparable to that reported in studies using phenobarbital, a widely used anti‐seizure medication (Auer et al. 2020). Although direct comparisons should be made with caution, the reduction in SRS duration achieved by astrocytic Igfbp2 deletion was similar to that observed with phenobarbital treatment.

Collectively, these results provide compelling evidence that increased expression of Igfbp2 in astrocytes exacerbates SRS parameters and actively contributes to the development of SRSs.

## 4 Discussion

In this study, we provide compelling evidence that Igfbp2 expression is upregulated in reactive astrocytes within hippocampal sclerotic regions (Figure 3), and that this elevation persists for at least 2 months following status epilepticus (Figure 4). Functional experiments demonstrated that treatment with Igfbp2 enhanced the excitability of dentate granule cells (Figure 5C), while selective deletion of Igfbp2 in astrocytes suppressed SRSs, which manifested as hippocampal paroxysmal discharges in our MTLE model mice (Figure 5J,K). Taken together, these findings suggest that astrocytic upregulation of Igfbp2 contributes to epileptogenesis.

Moreover, our RNA sequencing analysis revealed upregulation of BMP6 following KA administration, a known inducer of Igfbp2 expression in astrocytes (Caldwell et al. 2022). Consistently, we observed increased expression of phosphorylated SMAD (pSMAD), a downstream effector of BMP6 signaling, in astrocytes within the hippocampal sclerotic regions (Figure S3, *U* = 325.00, *p* = 5.95 × 10^−14^, Mann–Whitney rank sum test). Supporting the potential involvement of this pathway, one of the brain‐penetrant inhibitors of BMP–pSMAD signaling is Saracatinib, which was originally developed as a Src inhibitor (Kaufman et al. 2015; Williams et al. 2021). In pilocarpine‐induced TLE model mice, administration of Saracatinib for 14 days following status epilepticus attenuates the development of spontaneous recurrent seizures (SRSs) (Luo et al. 2021). These findings further support the notion that the BMP6–pSMAD signaling pathway in astrocytes may represent a potential therapeutic target for epilepsy. Our hippocampal sclerotic astrocyte‐enriched transcriptome data thus provide important insights into the contribution of astrocytes to epileptogenesis.

### 4.1 The Mechanism of Igfbp2 Involvement in Epileptogenesis

The dentate gyrus is widely recognized as a gatekeeper that restricts the spread of aberrant cortical activity into the hippocampus, and this gatekeeping function is considered fundamental to the emergence of SRSs in MTLE (Heinemann et al. 1992; Lothman et al. 1992). Recent studies have provided strong support for this “dentate theory.” For instance, optogenetic activation of dentate granule cells has been shown to trigger epileptic seizures even in naïve mice that had not been exposed to chemoconvulsive stimuli. Conversely, optogenetic or chemogenetic silencing of dentate granule cells in MTLE mouse models significantly reduced the incidence of SRSs (Krook‐Magnuson et al. 2015; Desloovere et al. 2019; Zhou et al. 2019).

We postulate the following three hypotheses regarding the role of Igfbp2 in epileptogenesis. First, Igfbp2 may act directly on neurons, altering their intrinsic firing properties. Second, Igfbp2 may facilitate the reorganization of neural circuits, thereby enhancing neuronal excitability. Third, Igfbp2 may increase neuronal excitability through neuroinflammatory pathways.

In support of the first hypothesis, our electrophysiological analyses demonstrated that Igfbp2 exposure led to enhanced granule cell activity. To evaluate whether this effect occurs within a physiologically relevant concentration range, we next estimated the amount of Igfbp2 present in the hippocampus and compared it with the concentration used in our experiments.

Based on ELISA data for Igfbp2 in the mouse hippocampus, along with total protein content and hippocampal volume, the estimated concentration of Igfbp2 in the normal mouse hippocampus is approximately 37–56 ng/mL (Khan et al. 2019; Chlebowski and Kisby 2020; Brait et al. 2021). According to our RNAseq data, Igfbp2 expression in astrocytes from the epileptic hippocampus is increased by approximately 11.5‐fold, suggesting that the total Igfbp2 concentration in the epileptic hippocampus may reach 370–560 ng/mL. Although the mechanism by which astrocytes release Igfbp2 remains unclear, BDNF, a molecule with a similar molecular weight, is known to be secreted via exocytosis. Under steady‐state conditions, cultured astrocytes release approximately 5% of their BDNF content (Han et al. 2021). Assuming a similar release ratio for Igfbp2, astrocytes in the epileptic hippocampus would be expected to release 18.5–28 ng/mL of Igfbp2.

In primary rat cortical neurons, JB2, a peptide fragment that mimics Igfbp2, dose‐dependently enhances intracellular calcium signaling (Burgdorf et al. 2023), suggesting that the neuroexcitatory effect of Igfbp2 is also dose‐dependent. Our in vitro electrophysiological data demonstrate that Igfbp2 at 10 ng/mL, a concentration that could be physiologically released, enhances the excitability of dentate granule cells. These findings imply that Igfbp2 may exert even stronger neuroexcitatory effects in the epileptic hippocampus, where its concentration could exceed 10 ng/mL. Another possibility is that Igfbp2 may indirectly enhance granule cell excitability via surviving pyramidal neurons in the CA3 region. Retrograde tracing studies have revealed an increase in CA3‐to‐dentate backprojections in the epileptic hippocampus of MTLE model mice (Zhou et al. 2019; Lybrand et al. 2021). As noted above, Igfbp2 has been shown to increase the excitability of pyramidal neurons (Khan et al. 2019). Thus, astrocyte‐derived Igfbp2 could elevate CA3 pyramidal cell activity, which may then be transmitted to dentate granule cells through these aberrant backprojections.

However, our current study does not elucidate the precise molecular mechanisms by which Igfbp2 enhances granule cell activity. Previous research suggests that Igfbp2 can bias the developing hippocampus toward hyperexcitability via IGF receptor signaling (Khan et al. 2019). Astrocytic Igfbp2 has also been shown to enhance hippocampal neuronal activity via IGF‐1R in P2Y1R‐overexpressing mice (Shigetomi et al. 2024). IGF‐1R is known to regulate neuronal excitability in several neuronal types. In the hippocampal CA1 region, neuronal IGF‐1R positively modulates synaptic transmission in pyramidal neurons (Gazit et al. 2016). Moreover, in small‐diameter trigeminal ganglion neurons, IGF‐1R reduces A‐type K^+^ currents, thereby inducing neuronal hyperexcitability (Wang et al. 2014). Studies by Khan et al. and Shigetomi et al. demonstrated that pharmacological inhibition of IGF‐1R acutely suppresses neuronal excitability. Given that the increased firing rate of dentate granule cells observed in our in vitro electrophysiological experiments also represents an acute response, it is plausible that the effect of Igfbp2 is mediated, at least in part, through IGF‐1R‐dependent signaling.

In support of the second hypothesis, given that Igfbp2 is a secreted molecule and its release from astrocytes has been associated with neurite outgrowth (Jeong et al. 2013; Caldwell et al. 2022), it is plausible that, in the epileptic hippocampus, astrocyte‐derived Igfbp2 promotes recurrent excitatory connectivity, such as mossy fiber sprouting, thereby increasing granule cell excitability. Following status epilepticus, synaptic density in the hippocampus declines sharply but gradually recovers to baseline levels during the later phase (Mikkelsen et al. 2022). During this period, the incorporation of immature granule cells into the dentate circuits might contribute to epileptogenesis (Jean et al. 2023). In the adult rat hippocampus and medial prefrontal cortex, Igfbp2 appears to promote spine maturation (Burgdorf et al. 2017). Whether similar or distinct molecular mechanisms underlie the effects of Igfbp2 in the epileptic hippocampus remains to be determined in future studies.

A third possible mechanism is that Igfbp2 exacerbates epileptogenesis by attenuating IGF‐1 signaling. The primary function of Igfbp2 is to bind and sequester insulin‐like growth factors, particularly IGF‐1. Notably, IGF‐1 has been reported to exert anti‐inflammatory effects during the subchronic phase in MTLE model mice (Wu et al. 2022). Given that neuroinflammation is increasingly recognized as a critical driver of epileptogenesis (Vezzani et al. 2011; Devinsky et al. 2013), we propose that astrocyte‐derived Igfbp2 may bind and sequester IGF‐1, thereby limiting its anti‐inflammatory action and facilitating epileptogenesis.

### 4.2 The Relationship Between Igfbp2 and Epileptogenesis, Ictogenesis

In epilepsy research, epileptogenesis refers to the process by which a previously non‐epileptic brain develops SRSs. This typically includes a latent period that follows an initial precipitating event and precedes the emergence of chronic seizures. In contrast, ictogenesis describes the acute, transient mechanisms that trigger individual seizures once an epileptic network has been established. These ictogenic events are often linked to momentary disruptions in the balance between excitatory and inhibitory signaling.

Our experimental design, in which AAV‐mediated SaCas9 was introduced prior to KA injection, was intended to assess the role of astrocytic Igfbp2 during the progression from a non‐epileptic to an epileptic brain state (Figure 5D). Thus, the study is fundamentally positioned to investigate epileptogenic processes. Supporting this, we observed a sustained upregulation of Igfbp2 in the KA‐injected hippocampus, detected by in situ hybridization and immunohistochemistry, persisting from early after the insult to at least 2 months post‐KA injection, a time point by which chronic epilepsy is established (Figures 3 and 4).

Notably, however, some of our outcome measures, specifically, seizure duration and severity, but not frequency (Figure 5I), are more traditionally associated with ictogenesis. Therefore, while our results clearly implicate astrocytic Igfbp2 in the establishment of epilepsy, they also suggest that it contributes to modulating the properties of individual seizures.

These findings are further reinforced by our acute slice electrophysiology data, which demonstrate that exogenous Igfbp2 increases dentate granule cell excitability. This supports a dual model in which astrocytic Igfbp2 facilitates epileptogenesis by enabling SRS emergence, and exacerbates ictogenesis by prolonging and intensifying seizure events.

To distinguish these temporal roles, future studies should adopt a strategy in which astrocyte‐specific Igfbp2 deletion is performed after epilepsy has already developed. Inducible Cre‐loxP–based systems would allow selective assessment of Igfbp2's influence on seizure dynamics independent of its role in disease onset.

Finally, a critical unresolved issue is the mechanism and timing of Igfbp2 release. Although astrocytic deletion of Igfbp2 significantly reduced seizure duration, it remains unknown whether this protein is secreted in response to neuronal activity during seizures, or in a tonic, activity‐independent manner. Recent data suggest that P2Y1 receptor–mediated calcium signaling, a canonical astrocytic response to neural activity, does not govern Igfbp2 secretion (Shigetomi et al. 2024). These findings raise the possibility that Igfbp2 is constitutively secreted, and future investigations will be essential to determine whether its release is dynamically regulated during ictogenesis.

### 4.3 Igfbp2 Expression in MTLE Patients

In evaluating the translational relevance of this study, it is important to determine whether Igfbp2 upregulation also occurs in patients with MTLE. A recent RNA‐sequencing study reported downregulated Igfbp2 expression in hippocampal tissue from MTLE patients (Dixit et al. 2016), which contrasts with our findings showing Igfbp2 upregulation in the KA‐induced epileptic hippocampus in mice. One possible explanation for this discrepancy is the choice of control samples in the human study, which were derived from glioma patients. Since Igfbp2 is known to be upregulated in glioma cells (Zumkeller et al. 1993; Dunlap et al. 2007; Phillips et al. 2016), the reported downregulation in MTLE samples may reflect a relative decrease compared to glioma tissue, rather than true reduction relative to healthy hippocampal levels.

Recent studies have deepened our understanding of astrocyte‐derived glutamate, a long‐standing controversial topic, and have also revealed the contribution of astrocyte‐derived factors such as GABA, l‐serine, and Serpina3N to the pathology of epilepsy (Pandit et al. 2020; de Ceglia et al. 2023; Liu et al. 2023; Sha et al. 2025). These findings underscore the significance of astrocyte‐derived factors in glia–neuron interactions under both physiological and epileptic conditions. Our study identifies Igfbp2 as a previously unrecognized astrocyte‐derived epileptogenic factor, which enhances dentate granule cell excitability and promotes epileptogenesis. While further studies are needed to directly assess Igfbp2 expression in human MTLE tissue using non‐tumor controls, elevated Igfbp2 levels have been observed in disorders with an increased risk of epilepsy, including Alzheimer's disease, multiple sclerosis, and gliomas (Zumkeller et al. 1993; Chesik et al. 2006; Hertze et al. 2014). These findings not only advance our understanding of MTLE pathophysiology, but also highlight astrocytic Igfbp2 as a potential therapeutic target in epilepsy and its related comorbidities.

## Author Contributions

R.K. conceived the study. S.K. performed most of the experiments, analyzed the data, and prepared the manuscript draft. N.M. conducted and analyzed the in vivo electrophysiological experiments. S.M. conducted and analyzed the in vitro electrophysiological experiments. Y.I. and R.K. acquired funding and contributed to project initiation. R.K. supervised the project and finalized the manuscript.

## Conflicts of Interest

The authors declare no conflicts of interest.

## Supporting information


**Figure S1:** Astrocytic Igfbp2 expression is also increased when comparing KA‐ and PBS‐injected hippocampi. (A) Volcano plot showing log_2_ fold change and –log_10_ adjusted *p* values for astrocyte‐expressed genes (FPKM > 1) comparing KA‐injected and PBS‐injected groups. Differentially expressed genes (DEGs) were defined using an adjusted *p < 0.05 and fold change > 2 cutoff. (B) Heatmaps showing FPKM values for the top 10 most altered astrocyte‐enriched DEGs (IP/input ≥ 2).

**Figure S2:** Transition of Igfbp2 expression in astrocytes after status epilepticus. Expression levels of Igfbp2 in immunoprecipitated samples (FPKM). *n* = 3 samples for Control and KA 14d group. *n* = 2 samples for KA 5d group. Data represent mean ± SEM.

**Figure S3:** Astrocytic pSMAD expression is increased in the hippocampal sclerotic regions. (A) Representative images showing Aldh1l1‐EGFP and pSMAD expression in the CA3 region of MTLE model mice. Scale bar, 50 μm. (B) Quantification of pSMAD fluorescence intensity in EGFP‐labeled astrocytes. *n* = 64 cells (contralateral) and 53 cells (ipsilateral) from 3 mice. Data represent mean ± SEM. ***p < 0.001, Mann–Whitney rank sum test.

## Data Availability

The data that support the findings of this study are available from the corresponding author upon reasonable request.
