# Supplementary figures and images for: Astrocytic Igfbp2 Promotes Spontaneous Seizures in a Mouse Model of Mesial Temporal Lobe Epilepsy
Source: Glia. 2025 Nov 15;74(1):e70099. doi: 10.1002/glia.70099 (PMC12619084; doi:10.1002/glia.70099)

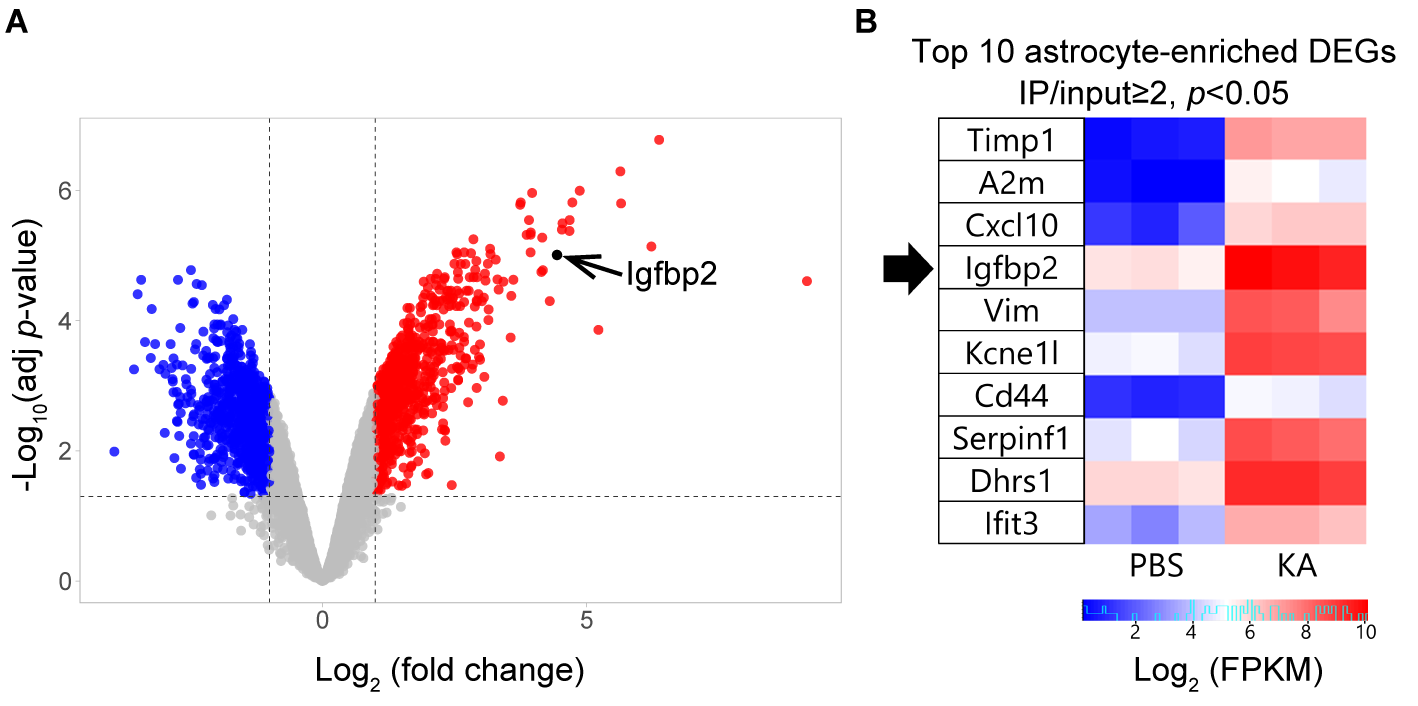

Supplement: Supplementary file 1 — Figure S1: Astrocytic Igfbp2 expression is also increased when comparing KA‐ and PBS‐injected hippocampi. (A) Volcano plot showing log2 fold change and –log10 adjusted p values for astrocyte‐expressed genes (FPKM > 1) comparing KA‐injected and PBS‐injected groups. Differentially expressed genes (DEGs) were defined using an adjusted *p < 0.05 and fold change > 2 cutoff. (B) Heatmaps showing FPKM values for the top 10 most altered astrocyte‐enriched DEGs (IP/input ≥ 2). [file GLIA-74-0-s001.tif]

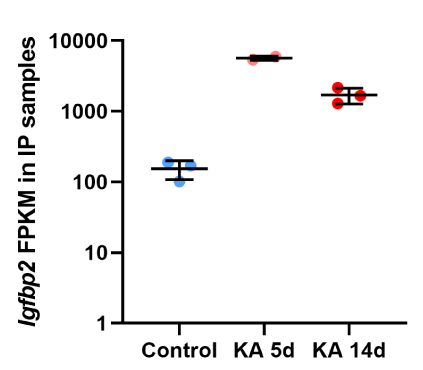

Supplement: Supplementary file 2 — Figure S2: Transition of Igfbp2 expression in astrocytes after status epilepticus. Expression levels of Igfbp2 in immunoprecipitated samples (FPKM). n = 3 samples for Control and KA 14d group. n = 2 samples for KA 5d group. Data represent mean ± SEM. [file GLIA-74-0-s002.tif]

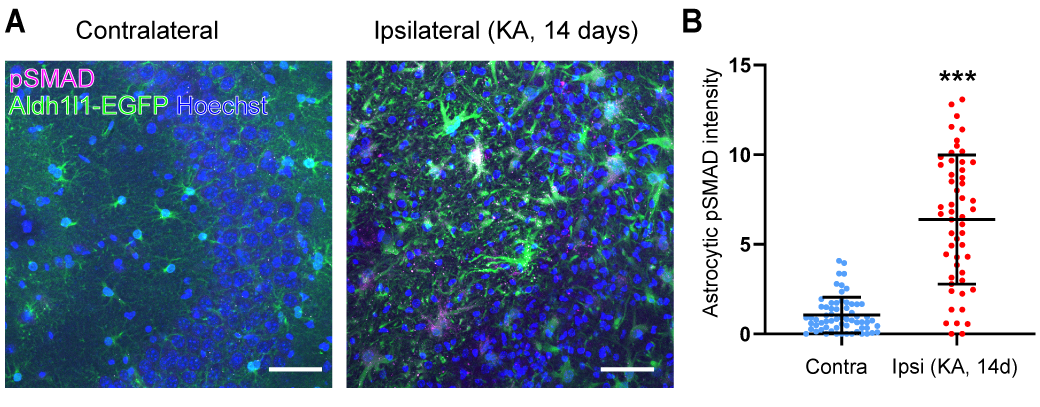

Supplement: Supplementary file 3 — Figure S3: Astrocytic pSMAD expression is increased in the hippocampal sclerotic regions. (A) Representative images showing Aldh1l1‐EGFP and pSMAD expression in the CA3 region of MTLE model mice. Scale bar, 50 μm. (B) Quantification of pSMAD fluorescence intensity in EGFP‐labeled astrocytes. n = 64 cells (contralateral) and 53 cells (ipsilateral) from 3 mice. Data represent mean ± SEM. ***p < 0.001, Mann–Whitney rank sum test. [file GLIA-74-0-s003.tif]
